# Radiographic Parameters in Predicting Outcome of Patients with Hepatocellular Carcinoma Treated with Yttrium-90 Microsphere Radioembolization

**DOI:** 10.1155/2013/538376

**Published:** 2013-09-15

**Authors:** Mohamed E. Salem, Nitin Jain, Gregory Dyson, Stephanie Taylor, Sherif M. El-Refai, Minsig Choi, Anthony F. Shields, Jeffery Critchfield, Philip A. Philip

**Affiliations:** ^1^Department of Oncology, Karmanos Cancer Center, Wayne State University, Detroit, MI 48201, USA; ^2^Department of Radiology, Wayne State University, Detroit, MI 48201, USA; ^3^University of Florida, Gainesville, FL 32611, USA; ^4^Section of Interventional Radiology, Department of Radiology, Wayne State University, Detroit, MI 48201, USA

## Abstract

*Background*. In patients with hepatocellular carcinoma, selection criteria for transarterial hepatic selective internal radiotherapy are imprecise. Additionally, radiographic parameters to predict outcome of transarterial hepatic selective internal radiotherapy have not been fully characterized. *Patients and methods*. Computed tomography (CT) scans of 23 patients with unresectable primary hepatocellular carcinoma before and after transarterial hepatic selective internal radiotherapy with yttrium-90 microspheres were retrospectively reviewed. Selected radiographic parameters were evaluated and correlated with progression-free survival and overall survival. Response to treatment was assessed with Response RECIST 1.1 and Morphology, Attenuation, Size, and Structure (MASS) criteria. *Results*. On the post-SIRT CT, 68% of tumors demonstrated decreased size (median decrease of 0.8 cm, *P* = 0.3); 64% had decreased attenuation (median decrease 5.7 HU, *P* = 0.06), and 48% demonstrated increased tumor necrosis (*P* < 0.001). RECIST-defined partial response was seen in 10% patients, stable disease in 80%, and 10% had disease progression. Median progression-free survival was 3.9 months (range, 3.3 to 7.3), and median overall survival was 11.2 months (7.1 to 31.1). Pretreatment lower hepatopulmonary shunt fraction, central hypervascularity, and well-defined tumor margins were associated with improved progression-free survival. *Conclusion*. In patients with unresectable hepatocellular carcinoma, pretreatment CT parameters may predict favorable response to SIRT and improve patient selection.

## 1. Introduction

Hepatocellular carcinoma is the third leading cause of cancer mortality worldwide [1]. It frequently occurs in patients who have chronic liver disease and cirrhosis [2, 3]. Curative treatment options include liver transplant, resection, and ablation [4]. However, approximately two thirds of patients are not candidates for curative therapy when diagnosed [5]. In selected patients, transarterial chemoembolization or radioembolization may be options for palliative treatment [6–9]. For patients with advanced hepatocellular carcinoma who are not candidates for curative or liver-directed therapies, targeted molecular therapy with sorafenib may improve survival [10, 11]. 

In the past, hepatocellular carcinoma had been considered a radioresistant tumor because of the limited effect of external beam radiation doses. This was caused, in part, by technical limitations imposed by the overlying anatomy [12, 13]. In selective internal radiotherapy (SIRT) with yttrium-90 microspheres, radioactive particles are injected into the hepatic artery, become trapped at the precapillary level, and emit lethal radiation to the tumor [6, 14, 15]. In SIRT with radioactive yttrium-90 microspheres, radiation exposure to normal liver parenchyma and extrahepatic structures may be limited, and higher radiation doses may be delivered to the tumor than feasible with external beam radiation. Nonetheless, the current patient selection criteria for SIRT are imprecise, and there is limited information available about the effect of SIRT on computed tomography (CT) characteristics of hepatocellular carcinoma. Furthermore, there is limited information about the relation between changes in CT characteristics and progression-free and overall survival after SIRT. Furthermore, it is not well studied whether CT scans can provide clinically useful objective parameters before or after SIRT in patients with hepatocellular carcinoma and whether the availability of these parameters may improve patient selection and treatment outcome from SIRT.

The purpose of this study was to characterize the radiographic parameters of hepatocellular carcinoma before SIRT with yttrium-90 glass microspheres; to evaluate whether these parameters may predict the outcome of SIRT; to characterize the objective changes caused by SIRT; and to evaluate the radiographic response of patients with hepatocellular carcinoma who were treated with SIRT with yttrium-90 glass microspheres. We characterized the features of tumors on CT scans before and after SIRT, assessed the radiographic tumor response by Morphology, Attenuation, Size, and Structure (MASS) criteria, and determined CT features that were associated with better progression-free and overall survival.

## 2. Materials and Methods

### 2.1. Patients

This study was a retrospective review of 23 patients who had hepatocellular carcinoma and who underwent SIRT with yttrium-90 glass microspheres between 2008 and 2011 at Karmanos Cancer Center, a tertiary care center. Inclusion criteria included (1) a diagnosis of hepatocellular carcinoma from biopsy or imaging according to criteria of the American Association for the Study of Liver Diseases [16]; (2) treatment with SIRT using yttrium-90 glass microspheres; and (3) survival >6 weeks after SIRT. Patients without proper follow-up imaging were excluded from the study. The study was approved by the Institutional Review Board.

### 2.2. Clinical Evaluation

The electronic medical records were reviewed for demographic, clinical, radiographic, and pathologic information before and after SIRT. Data included age, race, sex, hepatic function, renal function tests, liver Child Pugh status, imaging studies, and survival outcomes. Patients were staged by Child-Pugh score, United Network for Organ Sharing (UNOS) score, and Barcelona Clinic Liver Cancer (BCLC) score (A, early; B, intermediate; C, advanced; D, end-stage) [17, 18]. Patients survival was determined using the Metropolitan Detroit Cancer Surveillance System (MDCSS) or Detroit Surveillance Epidemiology and End Results (SEER) cancer registry. 

### 2.3. Selective Internal Radiotherapy with Yttrium-90 Glass Microspheres

The SIRT procedure was performed by percutaneous transarterial injection of radioactive glass microspheres (*β*-radiation; activity per microsphere, 2500 Bq; diameter, 20 to 30 *μ*m) into the hepatic artery. The microspheres were supplied in a V-bottom, shielded glass vial (range, 1.2 million to 8 million microspheres per vial); the number of microspheres determined the radioactivity of the vial (range, 3 GBq (81 mCi) to 20 GBq (540 mCi)). Radiation doses were customized for each patient by administering the beads at a defined time on the radiation decay curve. The SIRT dose was determined from CT-based liver volumetric analysis. Desired calculated doses per tumor-containing lobe ranged from 80 to 150 Gy [19–21]. 

The patients were evaluated with a CT scan and liver function tests before the SIRT procedure. Digital subtraction angiography was done with fluoroscopy to provide a map of the vascular anatomy, and vessels supplying extrahepatic structures such as the stomach or intestine were coil-embolized. A technetium-labeled protein was injected to determine the lung shunt fraction. Patients were excluded from SIRT when the hepatopulmonary shunt fraction resulted in a lung dose for a single session >30 Gy or cumulative lifetime >50 Gy. Patients had a second angiogram, and the radioactive beads were injected in a lobar fashion [19–21]. 

### 2.4. Computed Tomography Evaluation

The abdominal CT scans were performed before and after SIRT (Toshiba Aquilion 64 slice scanner, Toshiba Medical, Otawara-shi, Tochigi-ken, Japan or Siemens Somatom 64, Siemens, Munich, Germany). An abdominal radiologist who was blinded to the clinical outcome reviewed all CT scans. 

Primary tumor parameters were determined from the CT scans including (1) bidimensional tumor size; (2) tumor attenuation (density in Hounsfield units (HU)); (3) tumor margins (well or poorly defined); (4) tumor enhancement (homogenous or heterogeneous); (5) extent of tumor necrosis (<50%; ≥50% to <95%; or ≥95%); (6) hepatopulmonary shunt fraction; and (7) hypervascularity pattern (central or peripheral). The CT scans were also evaluated for the presence of portal venous thrombosis and lymphadenopathy.

The radiographic features of the primary tumor were determined on CT scans obtained at 3 times: before SIRT (baseline), after SIRT, and at the time of documented disease progression. The response of the primary tumor to SIRT was evaluated from the CT images using RECIST 1.1 [22] and MASS criteria [23]. The radiographic tumor responses by MASS criteria were categorized as favorable response, indeterminate response, or unfavorable response (Table 1) [23]. 

### 2.5. Data Analysis

Categorical data were reported as number (%). Measured data were reported as median (range, minimum to maximum). Categorical variables (tumor margins, enhancement, and percent necrosis) before and after SIRT were compared. The differences between continuous variables (tumor size and attenuation) before and after SIRT were dichotomized at the median for survival analysis. Changes between CT parameters before and after treatment were analyzed with Wilcoxon Signed-Rank Test (continuous variables) or Fisher's Exact Test (categorical variables). 

The primary endpoint of this study was progression-free survival (PFS), defined as the time from SIRT to disease progression (defined by RECIST 1.1 or death). The one patient who did not have a documented progression event was censored at the last evaluation for progression. The secondary endpoint was overall survival (OS), defined as time from SIRT until death. Patients who did not die were censored at the last time when they were known to be alive. Each of the categorical covariates was tested for association with progression-free and overall survival using the log-rank test. Continuous covariates were tested using Cox proportional hazards regression model. Median survival and 95% confidence intervals were estimated using Kaplan-Meier method. Bayesian analysis was applied to a multivariate Cox proportional hazards regression model to identify a parsimonious set of statistically significant covariates. Statistical significance was defined by *P* ≤ 0.05.

## 3. Results

Most patients were African American men who did not have ascites or extrahepatic metastases (Table 2). Baseline CT scans showed that most tumors had <50% necrosis, poorly defined margins, and heterogeneous enhancement (Table 2). 

Median tumor size was 8.1 cm (2.3–17 cm), median tumor attenuation was 35 HU (20–65.2 HU), and more than half of the tumors (52%) demonstrated central hypervascularity pattern. On the angiogram performed before treatment the median percentage of hepatopulmonary shunt fraction was 5.6%. The median dose of yttrium-90 glass microspheres administered to the patient was 2.0 GBq (0.6–4.3). 

Comparing the radiographic features of the CT scan obtained at median 1.5 months after SIRT to the CT scan obtained at baseline, most tumors margins were unchanged, and almost half of the number of tumors (48%) had increased percentage of necrosis (*P* < 0.001) (Table 3). Sixty-four percent of tumors demonstrated decreased attenuation (median decrease 5.7 HU, *P* = 0.06), and 68% of the tumors had reduction in longest diameters (median decrease of 0.8 cm, *P* = 0.3) (Table 3).

Following SIRT, the majority of patients (80%) had stable disease by RECIST 1.1. criteria; 10% of the patients demonstrated partial response, while 10% showed disease progression (Table 4). Only nine patients were response evaluable by MASS criteria; of theses 9 patients, 44% demonstrated favorable response while 22% and 33% of the patients demonstrated indeterminate and unfavorable response, respectively (Table 4). The median progression-free survival was 3.9 months (3.3, 7.3), and the median overall survival was 11.2 months (7.1, 31.1) for all patients.

Multivariate analysis of baseline CT parameters showed that prolonged progression-free survival was associated with lower hepatopulmonary shunt fraction, central hypervascularity pattern, and well-defined tumor margins, while shorter progression-free survival was associated with abutment of portal vein by tumor (Table 5). 

Subgroup analysis of the 9 patients who were response evaluable by MASS criteria showed that favorable response following SIRT was a predictor of overall survival (*P* = 0.027) (Table 6; Figure 6). 

Additionally, an exploratory analysis of the tumor size at base line (prior to radioembolization) showed that tumors >8.6 cm were associated with worse overall survival (HR: 1.92, 95% CI: (0.73, 5.04)), although the result was not statistically significant (*P* = 0.18).

## 4. Discussion

The therapeutic landscape has significantly evolved over the past decade largely because of a better understanding of tumor biology leading to the introduction of targeted therapies as well as technologies that revolutionized local-regional treatments. Yet, such advances have resulted in many challenges and raised several questions such as: which treatment modality is more potent and better tolerated? Can these treatment modalities be combined or applied sequentially? Lastly, how do we accurately assess the clinical efficacy? 

**Figure 1:**
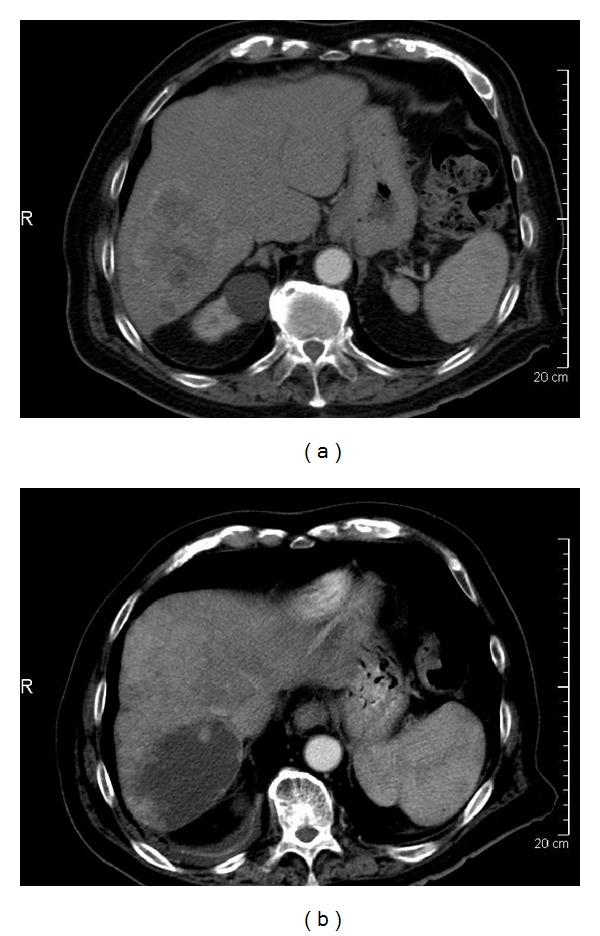
An 89-year-old man with hepatocellular carcinoma involving liver segment VI. (a) Before selective internal radiotherapy (SIRT), the tumor (10.2 cm) had poorly defined margins and peripheral enhancement. (b) After SIRT, the tumor had increased size (12.9 cm), increased necrosis, decreased vascularity, and decreased attenuation. This tumor was rated as progressive disease by RECIST 1.1 but favorable response by MASS criteria.

**Figure 2:**
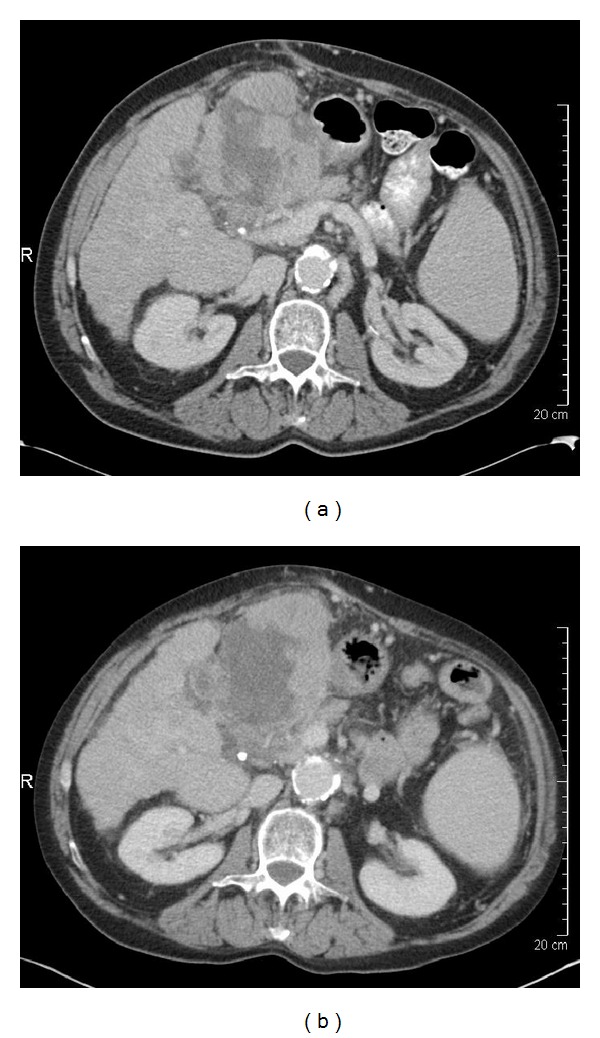
A 61-year-old woman with cirrhosis with multifocal hepatocellular carcinoma in liver segments II and III. (a) Before selective internal radiotherapy (SIRT), the tumor (13.3 cm) had central necrosis. (b) After SIRT, the tumor had minimal increase in size, increased necrosis, and decreased mean attenuation.

The antitumor activity of targeted agents, such as sorafenib or sunitinib, and the local treatment modalities, such as chemoembolization or radioembolization, may result in changes in tumor vascularization, cavitation, and necrosis that do not significantly affect tumor size. Consequently using RECIST criteria to evaluate tumor response after treatment may not predict patient outcomes well. For that reason, although RECIST has been an established tool for assessment of tumor response to conventional cytotoxic chemotherapy, its limitations in assessing the antitumor activity of certain liver-directed therapies and molecularly targeted treatments such as with antiangiogenic agents are increasingly recognized [24–27]. In fact, several studies have shown that response criteria based on size only may be an unreliable indicator of response to treatment [22, 28–31]. This is because of a phenomenon best described as “pseudoprogression” which is an increase in apparent tumor size possibly resulting from central necrosis, peritumoral edema, or intratumoral hemorrhage (Figures 1 and 2). This is perhaps due to the fact that these treatment modalities can reduce tumor vascularization, induce necrosis, and result in cavitations within solid tumors. Such changes may have no major effect on overall tumor size and frequently are read as stable disease or even progression by RECIST criteria. These patterns of tumoral changes have been reported in HCC and also with imatinib, sorafenib, and sunitinib use in other tumor types, such as non-small-cell lung cancer, renal cell carcinomas, and gastrointestinal stromal tumors [32, 33].

**Figure 4:**
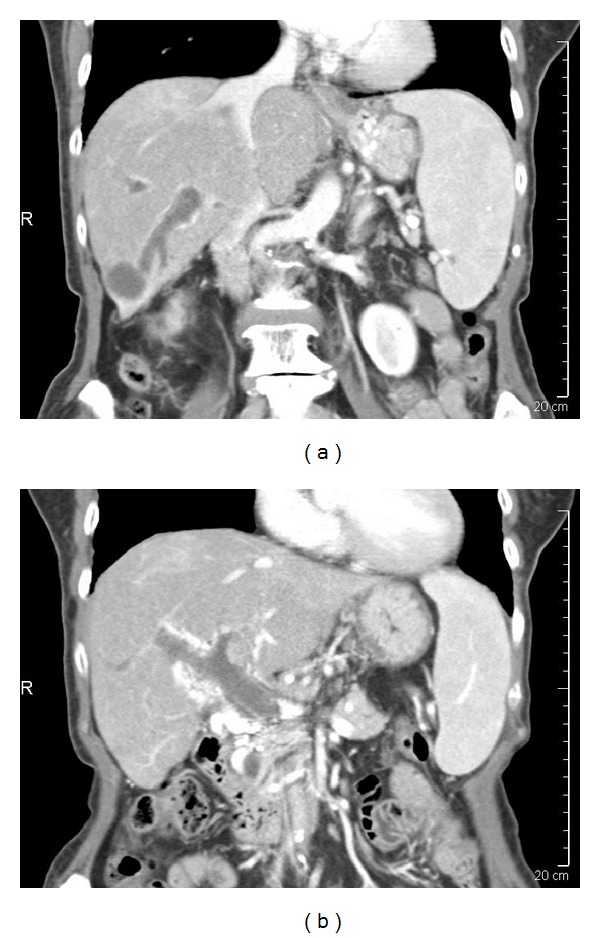
An 81-year-old woman with hepatocellular carcinoma. (a), (b) Before treatment with selective internal radiotherapy, tumor thrombus extended from the hepatocellular carcinoma (liver segment VI) to the right hemiportal vein (a) and main portal vein (b).

**Figure 5:**
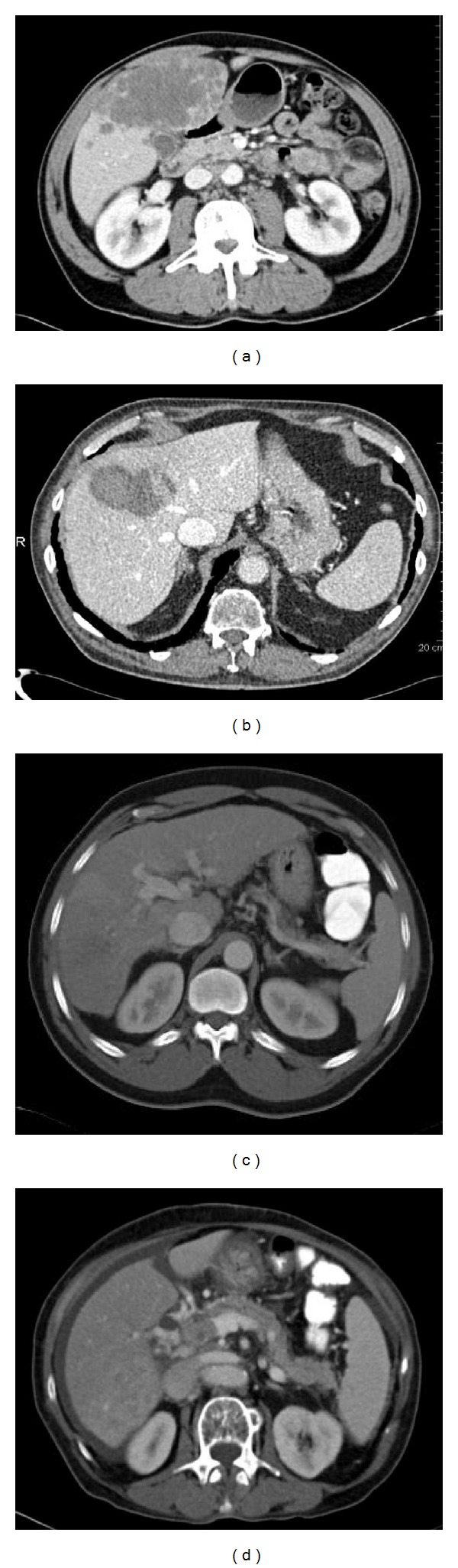
(a) A 63-year-old man with hepatocellular carcinoma. The right lobe of the liver was extensively involved with tumor, which had multiple satellite nodules and poor margins. (b) A 77-year-old man with hepatocellular carcinoma (6.4 cm) in liver segment VII that had well-defined margins. (c) A 59-year-old man with hepatocellular carcinoma that invaded the right hemiportal vein. (d) A 76-year-old woman with hepatocellular carcinoma and tumor thrombus in the main portal vein.

**Figure 6:**
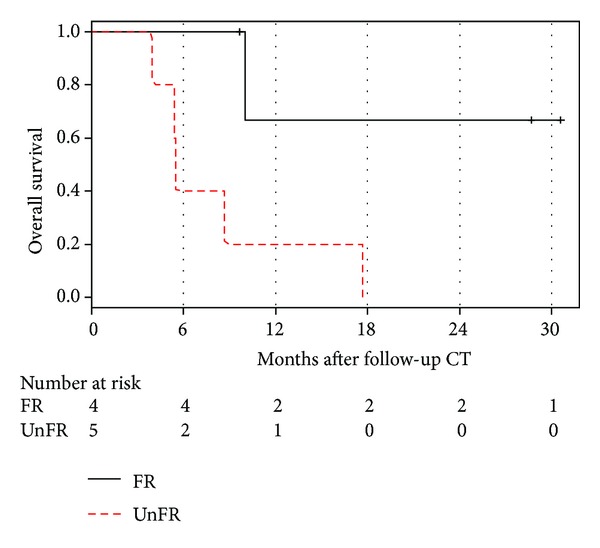
Kaplan-Meier plot showing overall survival stratified by Morphology, Attenuation, Size, and Structure (MASS) criteria (FR, favorable response; UR, unfavorable response).

In the present study we attempted to objectively quantify the radiographic tumor features on CT scans prior to treatment with SIRT. We hypothesized that CT images provided information on tumor size, tissue density, percent necrosis, hypervascularity pattern, margin irregularity, and portal vein invasion (Figures 4, 5(c) and 5(d)). This in conjunction with other tumor characteristics such as hepatopulmonary shunt fraction may enable the stratification of patients and improve selection of patients who may benefit from SIRT and serve as biomarkers in predicting response to SIRT.

In patients with hepatocellular carcinoma who were treated with SIRT, several CT characteristics of the primary tumors before treatment were associated with improved progression-free survival after SIRT (Table 5). Pretreatment features including well-defined tumor margins (Figures 5(a) and 5(b)), central hypervascularity pattern, and lower hepatopulmonary shunt fraction were associated with improved progression-free survival (Table 5). Therefore, these CT parameters may serve as biomarkers to distinguish patients who will respond to SIRT from those who may benefit from alternative treatment and be spared the potential toxicity and cost of SIRT. 

In addition, we evaluated the radiographic response following SIRT using MASS criteria to identify patients who favorably responded to radioembolization therapy versus those who needed additional therapy. 

After SIRT, there were no significant changes in tumor size, but a significant increase in the extent of tumor necrosis (as measured by enhancement) was noted (Table 3; Figures 1 and 2), and although it did not reach statistical significance, there was a suggestion of a decrease in tumor attenuation following SIRT (*P* = 0.06).

Collectively, these findings may explain why RECIST criteria alone are not a reliable indicator for objective tumor response with respect to evaluating response to radioembolization (Table 4). Conversely, the MASS criteria, which include additional radiologic tumor parameters such as morphology, necrosis, attenuation, and structure, were significantly associated with progression-free and overall survival. Thus, MASS criteria after SIRT may be better in predicting progression-free and overall survival compared with RECIST 1.1 (Table 6).

The results of this study are similar to previously described findings in patients with metastatic renal cell carcinoma [23]. A previous study of liver metastases from colorectal cancer showed that RECIST criteria and tumor density were less useful in assessing the response to yttrium-90 radioembolization treatment than ^18^F-fluorodeoxyglucose positron emission tomography/CT tomography [34]. 

Limitations of the present study include the retrospective design, post hoc analysis, and small patient population. However, the results may provide justification for a prospective trial to confirm and further evaluate the response to SIRT determined by MASS criteria. In addition, there may have been technical inconsistencies in CT scanning, subjectivity in CT scan interpretation, and variation in the time after SIRT for obtaining the CT scan after treatment. However, this may reflect realistic clinical practice situations. Additionally, the MASS criteria were initially developed in patients with renal cell carcinoma who were treated with tyrosine kinase therapy.

In summary, in hypervascular tumors such as hepatocellular carcinoma, imaging response criteria that account for changes in tumor morphology, percentage of tumor necrosis, attenuation, and size may be more sensitive to the antitumor effects of SIRT than criteria based on size alone. Additionally, certain CT parameters may serve as biomarkers to distinguish patients who will respond to SIRT from those who may benefit from alternative treatment. Future studies may include prospective investigation of the accuracy of MASS criteria as a possible predictor of primary tumor response after SIRT in patients with hepatocellular carcinoma.

## Figures and Tables

**Figure 3:**
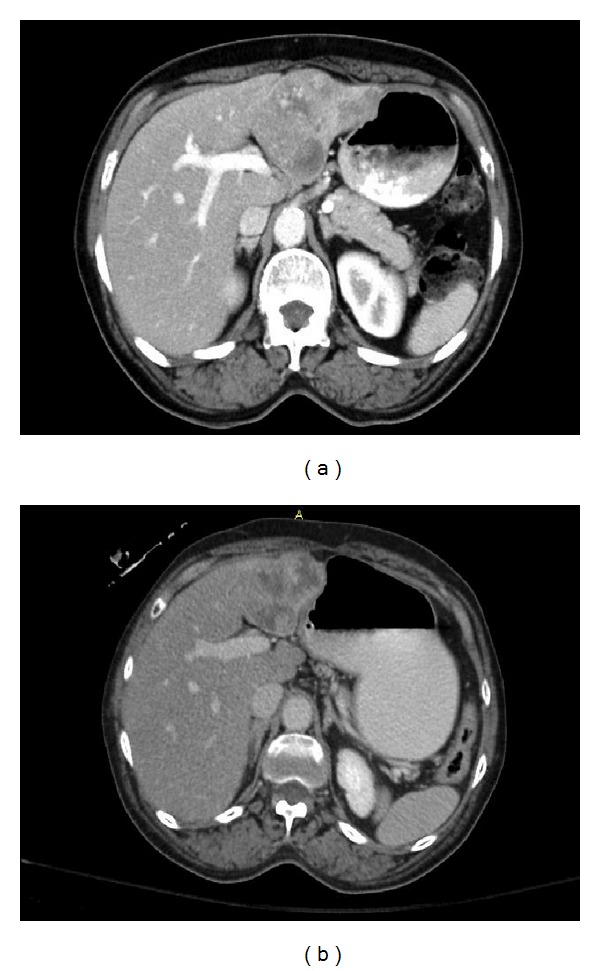
A 64-year-old patient with hepatocellular carcinoma. (a) Before selective internal radiotherapy (SIRT), tumor diameter was 8.4 cm. (b) After SIRT, the tumor had decreased size (6.7 cm; ≥20% decrease).

**Table 1:** Response to treatment graded by Morphology, Attenuation, Size, and Structure (MASS) criteria*.

Response category	MASS criteria description
Favorable response	No new lesions and either of the following: (1) decrease in tumor size ≥20%; (2) ≥1 predominantly solid enhancing lesion with marked central necrosis or marked decreased attenuation

Indeterminate response	Does not fit criteria for favorable or unfavorable response

Unfavorable response	Either of the following: (1) increase in tumor size ≥20% without marked central necrosis or marked decreased attenuation; (2) new metastases, marked central fill-in, or new enhancement of a previously homogeneously hypoattenuating, nonenhancing mass

*Described by Smith et al. [23].

**Table 2:** Characteristics of patients with hepatocellular carcinoma who were treated with selective internal radiotherapy with Yttrium-90 microspheres*.

Characteristic	
Age (y)	63 (50 to 87)
Sex	
Men	18 (79%)
Women	5 (21%)
Race	
African American	14 (61%)
White	6 (26%)
Other	3 (13%)
Ascites	5 (24%)
Extrahepatic metastatic disease	4 (17%)
Laboratory values	
Albumin (g/dL)	2.7 (2.7 to 4.9)
Total bilirubin (mg/dL)	0.8 (0.2 to 2.6)
*α*-fetoprotein (*μ*g/L)	108 (1.4 to 462400)
Computed tomography of tumors^†^	
Tumor size (cm)	8.1 (2.3 to 17)
Attenuation (HU)	35 (20 to 65.2)
<50% necrosis	21 (95%)
Well-defined margins	9 (39%)
Enhancement (heterogeneous)	21 (95%)
Central hypervascularity	12 (52%)
Hepatopulmonary shunt fraction	0.056 (0.014 to 0.165)
Peripheral hypervascularity pattern	6 (43%)

*N = 23 patients. Data reported as median (range, minimum to maximum) or number (%) patients.

^†^Before selective internal radiotherapy.

**Table 3:** Changes in computed tomography parameters of hepatocellular carcinoma after treatment with selective internal radiotherapy with Yttrium-90 microspheres*.

Parameter	Number (%) of tumors with change^†^	Median change	*P*
Decreased tumor size (*n* = 22) (Figure 3)	15 (68%)	−0.8 (−4.8 to +4.1) cm	0.3
Decreased attenuation (*n* = 11) (Figures 1 and 2)	7 (64%)	−5.7 (−14.8 to +6.1) HU	0.06
Increased percentage of necrosis (Figures 1 and 2)	10 (48%)	—	≤0.001
Margin status (*n* = 20)^‡^			
Improved	3 (15%)	—	0.2
Remained the same	14 (70%)		
Worsened	3 (15%)		

*Computed tomography was done at median 1.5 mo (95% confidence interval, 1.3 to 3.3 mo) after selective internal radiotherapy. Data reported as number (%) or median (range, minimum to maximum).

^†^Change from before to after treatment.

^‡^Improved, poorly defined became well defined; worsened, well defined became poorly defined.

**Table 4:** Response of hepatocellular carcinoma to treatment with selective internal radiotherapy with Yttrium-90 microspheres*.

Response criteria	No. (%) patients	*P* ^†^
RECIST^‡^		
Partial response	2 (10%)	0.09
Stable disease	17 (80%)	
Progressive disease	2 (10%)	
MASS criteria^¶^		
Favorable response	4 (44%)	0.027
Indeterminate response	2 (22%)	
Unfavorable response	3 (33%)	

^†^log-rank test; association of response criteria with overall survival.

^‡^RECIST: response evaluation criteria in solid tumors; *n* = 21 patients.

^¶^MASS: morphology, attenuation, size, and structure; *n* = 9 patients.

**Table 5:** Multivariate analysis of pretreatment computed tomography characteristics and progression-free survival in patients with hepatocellular carcinoma treated with selective internal radiotherapy with Yttrium-90 microspheres*.

Computed tomography characteristic	Hazard ratio (95% confidence interval)	*P*
Hepatopulmonary shunt fraction	0.28 (0.09 to 0.85)	0.02
Central hypervascularity pattern	0.13 (0.02 to 0.69)	0.006
Well-defined margins	0.37 (0.13 to 1.0)	0.04
Abutment of portal vein	10.1 (1.78 to 57.50)	0.002

*N = 23 patients.

**Table 6:** Multivariate analysis of response after treatment and overall survival in patients with hepatocellular carcinoma treated with selective internal radiotherapy with Yttrium-90 microspheres*.

Favorable response after SIRT^†^	Hazard ratio (95% confidence interval)	*P*-value
Overall survival	8.2 (0.93, 73.0)	0.027

*N = 9 patients. Response graded by Morphology, Attenuation, Size, and Structure (MASS) criteria.

^†^SIRT: selective internal radiotherapy. MASS criteria were not included in this multivariate analysis because of the small number of patients who could be evaluated by MASS criteria.
